# Health Without Care? Vulnerability, Medical Brain Drain, and Health Worker Responsibilities in Underserved Contexts

**DOI:** 10.1007/s10728-017-0342-x

**Published:** 2017-02-21

**Authors:** Yusuf Yuksekdag

**Affiliations:** Centre for Applied Ethics, Linköping University, Linköping, 581 83 Sweden

**Keywords:** Medical brain drain, Ethics, Vulnerability, Zimbabwe, HIV/AIDS, Health workers

## Abstract

There is a consensus that the effects of medical brain drain, especially in the Sub-Saharan African countries, ought to be perceived as more than a simple misfortune. Temporary restrictions on the emigration of health workers from the region is one of the already existing policy measures to tackle the issue—while such a restrictive measure brings about the need for quite a justificatory work. A recent normative contribution to the debate by Gillian Brock provides a fruitful starting point. In the first step of her defence of emigration restrictions, Brock provides three reasons why skilled workers themselves would hold responsibilities to assist with respect to vital needs of their compatriots. These are fair reciprocity, duty to support vital institutions, and attending to the unintended harmful consequences of one’s actions. While the first two are explained and also largely discussed in the literature, the third requires an explication on how and on which basis skilled workers would have a responsibility as such. In this article, I offer a vulnerability approach with its dependency aspect that may account for why the health workers in underserved contexts would have a responsibility to attend to the unintended side effects of their actions that may lead to a vital risk of harm for the population. I discuss HIV/AIDS care in Zimbabwe as a case in point in order to show that local health workers may have responsibilities to assist the population who are vulnerable to their mobility.

## Introduction

There is—with few exceptions [19]—a consensus that the effects of medical brain drain, especially in the Sub-Saharan African (SSA) countries, ought to be perceived as more than a simple misfortune [5, 7, 13, 21, 41]. Two critical aspects of medical brain drain from the SSA region are that nearly half of the trained health workers continuously emigrate from the already underserved region in order to work abroad and that the critical shortages exacerbate basic health delivery deprivation in the region [29: 68]. Temporary restrictions on the emigration of health workers from the region is one of the already existing policy measures to tackle the issue—while such a restrictive measure brings about the need for quite a justificatory work. A recent normative contribution to the debate by Gillian Brock [6] provides a fruitful starting point. As the first step in her defence of emigration restrictions, Brock provides three reasons why skilled workers themselves would hold responsibilities to assist with respect to vital needs of their compatriots and contribute to deprivation reduction in developing countries [6]. Notice that even if there are institutional and individual losses associated with brain drain, responsibilities for the skilled workers themselves to alleviate the deprivation are not automatically established. The developing states, thus, need a morally valid claim on skilled workers’ talents and assistance [36].

According to Brock, the first reason why skilled workers have a responsibility to assist is grounded in the virtue of fair reciprocity for the benefits they enjoy in the society, and more specifically for the state-funded tertiary education they have received [5, 14]. The second is that skilled workers should support the governmental efforts to meet the basic needs of the population, and they should not undermine their compatriots’ capacity to support legitimate governments that attempt to discharge their duties towards the citizens in good faith. The third is that skilled workers also have a responsibility to be attentive to unintended harmful side effects of their actions on other individuals. The idea is that all the reasons should be present for the skilled workers to have important responsibilities to assist with basic need satisfaction of their compatriots.^1^ This is an important first step in Brock’s argumentation that is imperative to the second step of deliberating permissibility of emigration restrictions. However, while the first two reasons are well described and explained, she does not explicate how and on which basis skilled workers would have a responsibility to attend to the unintended harmful side effects of one’s actions.

In this article, I offer a vulnerability approach with its dependency aspect that may account for why the health workers in underserved contexts would have a responsibility to attend to the unintended side effects of their actions that may lead to a vital risk of harm for the population.^2^ In addition to the background factors behind vulnerability of an individual to a risk of harm, dependency is an instructive yet often overlooked aspect of vulnerability which specifically deals with *to whom* the individuals are vulnerable to. The populations in underserved and resource-poor regions depend on the attentive assistance of health workers who can provide the much-needed care within the context of deprived health delivery systems that continuously lose their health workers. There is a noteworthy dependency relationship between health workers and population that the latter is *vulnerable to* the mobility of the former in a resource-poor and underserved area. From a normative perspective, despite the more descriptive approaches,^3^ this approach is an useful prism to discuss the individual responsibilities towards the vulnerable [17]. This normative approach warrants that health workers may have *some* responsibility to attend to the vulnerability and assist the individuals who are vulnerable to their actions and thus their mobility. I offer four criteria for such a responsibility to hold: the nature of the assistance requires a specific skill-set, the risk of harm should be vital and urgent, and the context should be underserved—while a shared history between the parties would also accentuate the responsibility. In addition, I point to responsibilities of the developing states to show good-faith efforts to alleviate the background factors of vulnerability—for a claim on health workers’ assistance on this basis to be fair and reasonable.

Notice that teasing out the reasons why health workers have responsibilities to assist the basic health needs of their compatriots is not sufficient to argue for a duty to stay, or to claim that restrictions are justified. Emigration restrictions, from a normative perspective, cannot be warranted solely on this ground. A balancing mechanism is needed to discuss (i) if the vital needs such responsibilities address outweigh the health workers’ right to exit, and (ii) the conditions under which the restrictive measures can be permissible.^4^ This article, therefore, focuses only on a small element of a much larger debate—by offering an approach which specifically addresses medical brain drain and explains why health workers in certain contexts may hold a responsibility to assist the individuals who are vulnerable to their actions.

The article has the following structure. In part 1, I introduce the vulnerability approach and its dependency aspect against which responsibilities for care professionals themselves can be argued. In part 2, I discuss HIV/AIDS care in Zimbabwe as a case in point. In part 3, I show that HIV/AIDS patients in Zimbabwe are *vulnerable to* the mobility of health workers due to (i) being in a resource-poor and underserved setting, and (ii) their dependency on the care of health workers—while the population might be especially dependent on the care of locally trained health workers given the nature of the much needed care and assistance for adequate healthcare provision. I contend that the local health workers may have responsibilities to assist the vulnerable in the given context. I conclude with the prospects of further research and policy-making.

## Vulnerability and Its Dependency Aspect

HIV/AIDS patients, elderly in home care, individuals deprived of means to subsistence, groups living in poverty-stricken environments, clinical patients with a certain degree of health illiteracy, and stigmatized research subjects are commonly referred to as vulnerable groups [16]. In the context of public health ethics, the concept is typically taken as a “marker for disadvantage” and as a “condition of constraint” through which subjects are more susceptible to ill-health (further ill-health and mortality) and unable to defend their vital interests due to background factors such as social determinants of health and lack of resources [35: 17, 38]. Most definitions, therefore, emphasize a wide range of aspects such as risk of harm incurred on individuals or groups, their vital interests and valid claims at stake; and the importance of capabilities and resources to cope with the harm. However, few discuss interpersonal dependency as an aspect of vulnerability [12, 24]. This will be done below.

Who then are vulnerable? Firstly, being vulnerable is being under an identifiable threat of harm to one’s vital interests or well-being, while lacking protection.^5^ In one sense, we are all vulnerable to ill-health. However, there are certain background factors and mechanisms, e.g. epidemics and social determinants of health that render individuals more susceptible to a certain harm. Imagine a child suffering from the symptoms of HIV/AIDS in the SSA region. Access to certain diagnostics could enable her to start the therapy right away and decrease the likelihood of mortality. She is also from a resource-poor family living in a rural area. The closest physician is hundreds of kilometres away and the family cannot afford such a trip. In addition, fear and stigma related to the disease might alienate the family from the rest of the community. The child like everyone affected by HIV/AIDS is vulnerable because of the disease. On top of that, poverty, stigmatization, health illiteracy and most importantly lack of care are additional factors which render her more vulnerable. As Florencia Luna and Samia A. Hurst assert, background factors of vulnerability can be additive or aggregative to the extent that different mechanisms and factors make an individual more susceptible to a certain harm, in this case ill-health and mortality [22, 27].

Secondly, her vital interest in adequate healthcare provision is also *vulnerable to* the actions of the physicians who hold the distinctive skill-set to diagnose the disease. To some extent, we all depend on care professionals and their specific skill-sets that makes us vulnerable to their actions. However, there might be certain background factors or circumstances through which agents become especially vulnerable to certain actions and omissions of particular agents. What if the physician, on top of all the other factors, leaves the public health facility? Within the context of abovementioned background factors, the child would be especially vulnerable to mobility of the physician as in the short-term there is no one to substitute the physician whom she depends on to access the much-needed and vital health care. All in all, being vulnerable should also be understood as being susceptible to harm due to certain actions or omissions of particular agent(s) who holds a distinctive capacity to alleviate/cause the threat of harm, ill-health and mortality [17: 111–112].

It is important to briefly explicate the relationship between dependency and vulnerability. As Susan Dodds [12] points out, one might be vulnerable—yet the degree of dependence on the assistance of a particular agent might vary. There might be vulnerable individuals who are dependent on the assistance of particular individuals because of a specific skill-set or a relationship, and there might be vulnerable individuals who are not dependent on the assistance of particular individuals. For the latter, vulnerabilities are rather the targets of structural and institutional interventions than personal care as for the many background factors represented above [12: 183]. Dodds exemplifies the difference by asserting that while all octogenarians are vulnerable to ill-health, not all of them are dependent on personal caregivers. Besides, whether or not an octogenarian becomes dependent on a caregiver might also be contingent upon a variety of background factors, such as one’s genetic faculties, socioeconomic status, and the provision of welfare support. Therefore, vulnerabilities can be divided into dependence and non-dependence vulnerabilities, whereas the line between them is not always crystal clear [12: 184].

I would also like to dispute the stigmatized nature of dependency relationships here [28]. I do not consider dependency as a weakness, but rather as a structural factor of our interpersonal relationships. Dependency is a component of basic human relationships rather than something to eliminate unlike poverty [15]. Second, dependency incorporates a variety of goods human beings enjoy. Responsiveness to the needs of the others, personal connection, attentiveness, and concern for the well-being of others are among the significant goods embedded in the practice of health care [24]. Care provided by a health worker also accommodates, or should accommodate all of these important social goods. In the context of public health where the care practice is an important component, there is a merit in highlighting the interpersonal dependency aspect of vulnerability. However, we should also recognize that certain background conditions render individuals especially vulnerable to the certain actions and omissions of particular agents—as in the example of the child. The responsibility for the latter falls to the hands of the developing states to address, yet both aspects are paramount when discussing why certain care professionals have responsibilities to protect the vulnerable in the context of underserved areas.

### Vulnerability Approach and the Basis of Individual Responsibilities to Assist

The idea that specific individuals have responsibilities towards the ones who are vulnerable to their actions can be traced back to Robert Goodin’s [17] welfare consequentialist account. Goodin suggests that individuals bear special responsibilities for protecting the interests of other individuals who are dependent upon and particularly vulnerable to their actions and choices [12: 189, 17: 33]. The emphasis is on the dependency of individuals on the actions and omissions of others; and the extent to which that leaves individuals helpless in the face of harm. So, principally for Goodin, the responsibility falls to the individual or the group of people who is in a special position (based upon a specific skill set or capacity to affect) to protect the vital interests of the vulnerable [17]. A filial relationship is one example of such dependency—as the dependency of the elder parent on her adult child is the reason behind one party being vulnerable to the actions of the other. Special responsibilities of adult children towards their elder parents can be explained by appealing to this morally significant relationship and by pointing to vulnerability of elder parents to actions and omissions of their adult children [44: 66]. However, this responsibility is not self-evident in the sense that it does not explain why adult children would have “larger than average responsibilities” to provide an extensive care to parents [44: 66].^6^

Vulnerability approach requires a little more scrutiny here to specify *why* certain agents would have a responsibility to avoid an identifiable harm. It has been suggested that the mere presence of dependency does not justify any responsibility to provide an extensive care on the part of adult children. The objection is that there might be others who can provide care for the elder parent in question [44: 66]. However, elder parents depend on their adult children in order to access many emotional values such as trust, appreciation, enjoyment, esteem and security that can be best provided by their adult children [45: 98]. The distinct personal capacity of the children can be pointed out that elder parents depend on their children to satisfy their emotional needs. In addition to that the shared history of the parent and the child strengthens the basis for responsibilities of the children towards their elder parents. Regardless, one can still claim that none of these reasons do justify any extensive responsibility to care, but to maintain the relationships by keeping in contact and paying visits [44: 66]. Certain background factors, nonetheless, can account for why certain agents have a responsibility to provide an extensive care to the individuals who are vulnerable to their actions. Take the example of a dependency relationship based on a professional skill-set within a context where there is no one else to help but one. Suppose that I am having a heart attack on a plane where there is only one physician. I am vulnerable to actions or omissions of this physician that I travel together with as my vital interest depends on his or her urgent care at the very moment. This would point to a responsibility on the part of the physician to alleviate the risk of harm, even if there is no distinct personal or emotional capacity or a shared history at play [48]. The conventional acceptance of the physician’s responsibility to assist points to the moral emphasis we ascribe to two present reasons that can additionally account for why certain agents have responsibilities towards the vulnerable: being vulnerable to a vital and urgent risk of harm, and being dependent on a particular caregiver in an underserved context.

In the end, three necessary criteria can be given to point out when and why certain agents would have a responsibility to protect or assist the vulnerable. The first is that an agent has a distinct capacity or skill set to prevent the risk of harm. The distinct capacity can take the form of a professional skill-set to diagnose and address a disease, and it can also accommodate a locally relevant, cultural or personal knowledge or capacity which would address both material and emotional needs of the vulnerable. Therefore, depending on the nature of the required assistance and care, responsibility can be based on a professional and/or an individual dependency relationship. Notice that a professional care relationship might also contain many personally and culturally relevant goods which are essential to the practice of care. In many clinical cases, trust, attentiveness and cultural literacy are paramount to a well-working patient and health worker relationship. The second is that the context should be underserved. There should be, putting it roughly, no one else in the area to exercise this distinct capacity or skill-set. Third is that what is at stake is a vital and urgent interest such as ill-health and mortality. Additionally as a fourth criterion, there might be a shared history between the caregiver and the vulnerable that relates them in a morally significant way which would strengthen the basis why some agents should have “larger than average” responsibilities [44: 66]. Taking the example of the child with the symptoms of HIV/AIDS as an illustration, responsibilities of a physician can be pointed out—when an agent (the child), especially depends on the care of another agent (the physician), who has a distinct capacity or skill set to prevent a vital harm (mortality or ill-health), in a certain context (resource-poor and underserved region). In addition, a shared history would also strengthen the basis of a care professional’s responsibility to assist the vulnerable. This line of reasoning addresses morally urgent needs and interests, and more importantly, it provides the basis why and when certain agents, the physician in this case, would have a responsibility to prevent the risk of harm towards the individuals who are vulnerable to his or her actions.

### Complementary Responsibilities of the States Within Resource-Poor Settings

The vulnerability approach still requires a little more scrutiny here in order not to disregard the responsibilities of the developing states to avoid an identifiable risk of harm. While, an individual health worker might hold a responsibility to acknowledge the dependency and assist her compatriots; the developing state has a responsibility to prevent and alleviate the background factors of vulnerability. Institutional and structural interference is a must to address background factors such as uneven resource allocation and corruption which lead to deprived and underserved health delivery systems, or other conditions such as health illiteracy and poverty which render an individual more susceptible to contagious diseases and further ill-health. The developing states should also take all the necessary, yet possible, measures to address push factors and to motivate voluntary retention.^7^ However, this does not negate the individual responsibilities, especially in the case of longstanding emergencies in the short-term. It is likely, as Brock suggests,^8^ that even if the developing states show good-faith efforts to sustain their vital institutions that address the basic needs of their citizens, mass exodus of human capital, especially in the short-term continues to be detrimental to implementation and viability of such efforts [6].

I contend that for individual health workers to hold responsibilities on the basis of vulnerability, the developing states should also fulfil their responsibilities and at least show good-faith efforts in sustaining their vital institutions. It is not only intuitively problematic, but also an unfair burden-sharing if the emigration leads to unintended harms solely on the basis of inadequate governmental intervention [23]. Nevertheless, the developing states are in a non-ideal situation, and this should be recognized in order not to expect financially and politically demanding interventions by resource-poor countries.

## Medical Brain Drain in Resource-Poor and Underserved Contexts: HIV/AIDS Care in Zimbabwe

How does the notion of vulnerability contribute to the understanding of medical brain drain^9^ from underserved regions as the SSA and why would it pinpoint to individual responsibilities, specifically for health workers? At the outset, the number of health workers necessary for adequate care is insufficient in the SSA countries [47: 120–127]. While the SSA countries need to increase their health workforce by 140% in order to meet the health-related Millennium Development Goals, around 30% of health workers from these countries emigrate for better job and living opportunities in the developed countries [25]. This leads to diminishing quality of care in the region struck by poverty, diseases, and epidemics. The more health workers the region loses, the less able they are to cope with the ill-health and mortality as they are dependent on the care of the remaining health workers.

Christine Straehle has recently demonstrated the relevance of vulnerability for medical brain drain [43]. She has introduced the lens of vulnerability in understanding the morally relevant factors pertaining to the emigration of nurses in the case of Malawi. Straehle [43] argues that a wrong occurs when pregnant women suffer from ill-health and mortality only because necessary employable means to prevent harms are not accessible. What is morally salient here is that certain constraining background factors such as “lack of detection, prevention and treatment” generate this vulnerability [43: 255]. Straehle refers to the general poor state of health care system as the background factor of vulnerability in Malawi. So that the emigration of nurses *aggregates* the vulnerability of the population to the extent that it is detrimental to preventative and therapeutic measures.

Rather than problematizing the background factors alone, this article aims at emphasizing particularly *to whom* individuals are vulnerable to. This points to the significance of dependency relationship between health workers and patients—a relation that ought to be acknowledged within the context of a resource-poor and underserved area, in addition to tackling the background factors of vulnerability. It is true that we are all dependent on care, responsiveness, trustworthiness and attention of health workers, while being vulnerable to their actions. What makes the medical brain drain from the SSA severely problematic is that given the context of exacerbating background factors and deprived health delivery systems, despite the governmental efforts, the population is vulnerable to the international mobility of health workers as their vital interest in an attentive health care depends *particularly* on the care of health workers in question. There might be also certain cultural features of the needed assistance and care that can be only addressed by the locally trained health workers. Therefore, depending on the context, the nature of the much-needed assistance and care provided by health workers should be explicated.

To substantiate this line of reasoning, in the following section, I exemplify how medical brain drain is a challenge for HIV/AIDS patients in Zimbabwe despite the relatively efficient implementation of antiretroviral therapy (ART) by the governments. I claim that this is due to high level of dependency of the HIV/AIDS patients on care provided by the health workers for adequate healthcare provision within the ART program. The case of Zimbabwe is relevant for two reasons: First, despite the successful preventative measures and progressive expansion of health sector as a result of the governmental efforts, Zimbabwe still has one of the highest prevalence of HIV/AIDS rates in the region. HIV/AIDS prevalence rate was estimated approximately at 14% in 2008 and the epidemic was responsible for approximately 15% of all deaths in Zimbabwe the same year [1]. Second, implementation of ART in Zimbabwe has declined mortality rates over the years, while the country still suffers from severe medical brain drain. Implications of the physician emigration on the efficiency of ART warrants a closer look.

### Background Considerations: Burdens on HIV/AIDS Patients in Zimbabwe

About 1.1 million Zimbabweans live with HIV/AIDS, and the country has one of the most severe HIV/AIDS epidemic rates in the region. The question is, to what extent emigration of physicians puts extra burdens on HIV/AIDS patients? The shortage of health workers in public hospitals overburdens the staff in the form of long working hours and poor working conditions. In a resource-limited setting with lack of protective medical equipment, e.g. gloves, a fear of contracting the disease also may make the health workers reluctant to provide care for HIV/AIDS patients. These aspects have been push factors behind emigration of health workers in the last decade [11]. Private medical institutions and NGOs have also been centres of attraction for public health workers and medical teaching staff in the country. Despite policy responses, such as short-term deployment of foreign experts, soft bonding measures towards graduates, extra allowances to local health workers, uncompetitive compensation in the public sector is still the major obstacle of sustaining an adequate number of health workers within both local and global job market [10: 677, 33: 100].

However, with the joint efforts of WHO and National AIDS Council of Zimbabwe, the introduction of the ART program has proven feasible and effective for HIV/AIDS patients. ART is a treatment consisting of the combination of at least three antiretroviral (ARV) drugs to maximally suppress the HIV virus and stop the progression of the disease. Zimbabwe reduced HIV/AIDS-related annual deaths from approximately 123,000 in 2006 to 71,299 in 2010 [49: 10]. In 2012 approximately 80% of eligible HIV/AIDS patients were covered by the ART program. Certain therapies combined with ART are provided to reduce mortality rates due to other complications and infections such as tuberculosis. Identification of HIV status is a must for a proper therapy.

Provision of ART requires sustainable care including HIV counselling, prevention, testing, diagnosis and treatment. Most importantly adherence to treatment is crucial as viral resistance to ARVs may emerge. For ART to be effective, a continuous and strict monitoring should be provided by physicians. Effective management of the disease thus implies a chronic patient care [33: 100]. Almost 600,000 individuals are in need of ART in Zimbabwe and scale-up is vital for the future prospects of public health, while even the routine health care is at stake due to the shortages.

ART scale-up, in the midst of medical brain drain, is no easy task. There is a need for an additional 1–2 physicians to be able to provide ART to an additional 1000 patients [20: 4–16]. Given that physician density per 1000 population is 0.067 in 2009, emigration of health workers proves quite problematic for the prospects of the therapy. This is particularly marked in rural areas where physician density can be as low as 0.02. Only the cities of Bulawayo and Harare are able to meet the benchmark of Millennium Development Goals [1]. Besides, even without the ART program, HIV/AIDS highlights the need for health workers in the face of complications and opportunistic infections such as tuberculosis [30].

On top of these challenges, poverty is a detrimental background factor for adequate healthcare delivery. Access to ART is in the first place problematic for low-income households. Food insufficiency, socio-economic stressors and high costs of transportation to the ART centres are all among the other factors pertaining to the low adherence to ART [18, 32, 40]. Even a simple process of transportation to the ART centres, in order to access ARVs is a big challenge for the community especially in rural areas [31].

Quality of care is another substantial component of ART. Patients need attention addressing their social and emotional needs throughout the therapy. Caring transcends mere accessibility. It also has a personal component. Patients still need caregiving of relatively high quality during the ART program to reach high adherence rates [46: 53–56]. Patients, especially in rural areas, suffer from long waiting hours, lack of care, and social discrimination in some cases. Lack of knowledge on precautionary measures towards the threat, health illiteracy, ineffective traditional responses, and stigmatization of HIV/AIDS patients also put a huge burden on the prevention and diagnostics. Additionally, the epidemic and the critical shortage have increased the workload of health workers especially in the public sector while creating burdens for both caregivers and patients. Health workers in Zimbabwe find caring for HIV/AIDS patients particularly stressful compared to other forms of care work, a factor that might result in patients receiving poor quality of care [10: 674]. Foreign health workers can relieve the burdens on the local staff. Nevertheless, given the stigmatized nature of the disease, cultural and linguistic differences may affect the quality of care. An agreement between the Zimbabwean and the Cuban governments in 2000 has led Cuban physicians to practice in the country. However, language barriers seem to lead to poor communication [10: 676]. Cultural and linguistic differences in addition to low health literacy may therefore render the patients more vulnerable to harm [26, 39]. In brief, follow-ups and sustainability of attentive and personal care by health workers are of utmost importance for the effectiveness of the ART program in Zimbabwe. Health workers, nevertheless, emigrate due to many push and pull factors such as relatively low remuneration and recruitment practices through which the country cannot compete with the relatively poor resources available to the government. The policy responses seem unable to address the shortages.

## Health Without Care? The Basis of Responsibilities to Assist HIV/AIDS Patients in Underserved and Resource-Poor Contexts

To what extent and on which basis is the Zimbabwean population dependent on the care of the health workers and thus vulnerable to their mobility, and to what extent this relationship points to responsibilities of health workers to assist the vulnerable? In the case of HIV/AIDS care in Zimbabwe, poverty is a crucial background factor. Secondly, within this context, dependency of the patients in the ART program on attentive and continuous care of the physicians render the population vulnerable to the mobility of the health workers.

### Vulnerability in Zimbabwe: Poverty and Dependency on Care

*Poverty* is one of the background factors of vulnerability. As a social determinant of health, poverty firstly works as a threat of harm to health. For instance, lack of nutrition, other forms of deprivation (e.g., lack of sanitation and water) and health illiteracy do diminish the public health delivery. Secondly, poverty is also a factor which makes the populations unable to cope with the harm. In addition to lack of equipment and poor health delivery infrastructure, substituting migrant health workers, or training new health workers is a limited capacity in the given context. These concerns can be exemplified with the ART program that is in need of more health interventions. Medical brain drain from Zimbabwe is not declining in the near future considering the steady flow of trained health workers [29]. The gap between the resource-rich and resource-poor countries in attracting health workers is unlikely to wither away in the near future. In addition to that, the unequal distribution seems to get aggravated by active recruitment policies of the developed states and private recruiting agencies. On top of push factors, admission policies of affluent states favouring the skilled labour facilitates the process in general [23: 1108]. Public health institutions in Zimbabwe seem unable to retain their health workers, while poverty endangers an effective implementation of ART for individuals lacking nutrition, means of transportation and medical equipment.

The second aspect of vulnerability is *dependency on care.* Prognosis and monitoring by health workers is vital for the health prospects of the population as patients in the ART program rely on an attentive care of health workers. The *nature* and *extent* of care of health workers presents itself even as particularly demanding form of care here in the context of the ART program in which repeated health worker interventions is a must. The more extensive the dependency is, the greater the importance of personal, reliable, and attentive care becomes [12: 184]. If the recruitment policies also target the senior physicians in the region, the quality of care would severely diminish along with the quantity. Due to reasons of poverty and health illiteracy, underprivileged members of the resource-poor country also become vulnerable to actions and omissions, and more specifically the mobility, of the health workers in an underserved context.

### Dependency on Local Health Workers

As outlined above, adequate and continuous care^10^ is essential for HIV/AIDS patients. Furthermore, adequate healthcare provision is contextually embedded to a certain extent. Shared language and cultural awareness are important conditions for trust as are interpersonal dialogue and attentive care [2–4]. For instance, in a case study within the context of HIV care in the US, “cultural distance was associated with lower patient ratings of healthcare provision quality and less trust in healthcare providers, along with lower levels adherence and viral suppression” [37: 282]. Trust, interpersonal dialogue and a certain cultural proximity are among the values an adequate care provision should address. Therefore, if there is something about the nature of the much needed care which specifically points to dependency of the population on the local health workers, this should be disclosed.

Foreign health assistance to improve the ART programs may prove useful. However, given the prevalence of health illiteracy, stigmatized nature of HIV/AIDS, and the necessity of chronic and constant care, an effective and perpetual patient caregiver communication come into prominence. The ways in which health illiteracy can be addressed requires a certain contextual and cultural knowledge and awareness. In addition to that, if the nature of the assistance necessitates an urgent attention and care, proximity can also be taken into account. Local health workers are thus in a special position to provide more effective (if not the best) care. There are also some practical concerns regarding foreign health assistance in the form of temporary exchange and residency programs. While the region is losing its qualified health workers, the developed countries might tend to send their graduates to acquire their residency status. This is a stage in which the likelihood of medical complications and errors leading to ill-health and mortality is significantly higher in clinical and operational care [34]. Therefore, locally and culturally relevant nature of the much-needed care points to the necessity of local health workforce assistance. Furthermore, there might be a shared history in between the physicians and the population which bind them in a morally relevant way. One might question when and how such a shared history comes about in between the local health workers and the population. What if a medical graduate wishes to emigrate right after the graduation without having any contact with the patients? Medical trainees, however, can be already considered to have a shared history with the population during their training, as they already get involved with many patients during their professional training—a period in which they operate on the patients with limited knowledge [14].

One can conclude that the drain of care is very problematic for the HIV/AIDS patients, and there is a basis for acknowledging the dependency relationship between local health workers and HIV/AIDS patients in Zimbabwe that may render the latter vulnerable to the mobility of the former. I contend that the local health workers themselves have a responsibility to assist the vulnerable considering the longstanding good faith efforts of the government. This line of reasoning is based on four criteria. The first is that the context is an underserved one as it already suffers from critical shortages that contributes to basic health delivery deprivation. The second is that what is at stake is ill-health and mortality of the population that require the assistance of health workers. The third is that the HIV/AIDS patients in Zimbabwe do depend on long term and attentive care of health workers. In addition, local health workers also have a distinct capacity to help as there are some locally and culturally embedded aspects of the much-needed care along with some practical advantages. The fourth is that there is already a certain shared history in between the local health workers and the population.

## Conclusion: Implications for Further Research and Policy-Making

In this article, I offered a vulnerability approach with dependency aspect that highlights why local health workers themselves have a responsibility to attend to the vulnerability and thus assist the individuals who are vulnerable to their actions. In addition to good-faith efforts of the developing states to sustain the basic health delivery in the region, I provided four criteria for why health workers themselves have responsibilities on the basis of unintended consequences of their mobility. This approach fills the gap that exists in Brock’s argumentation. It can also be interpreted as another basis why health workers, more specifically, would have responsibilities themselves to contribute to health delivery deprivation reduction in the developing countries.

In conjunction with the other reasons why health workers have a responsibility to assist, the second step would be then providing a balancing mechanism to discuss if vital needs such responsibilities address outweigh the health workers’ right to emigrate.^11^ An answer to this question requires (i) providing an account of the right to emigrate, and (ii) the conditions under which the restrictive measures can be permissible.^12^ Brock does not provide an account of the right to exit nor specify a balancing mechanism on that regard. This warrants a further work if one aims at justifying temporary restrictive measures on the basis of medical brain drain in developing countries, along with addressing feasibility and viability of such restrictive measures.

## Footnotes

1 Notice that while the responsibilities with respect to compatriot deprivation are to be discharged via the state apparatus, they stem from the moral significance of basic and vital needs of the populations [8].

2 Christine Straehle [43] is the pioneer in this regard, and I aim to provide a nuanced inquiry. I briefly introduce her account in the beginning of the second part of this article.

3 By descriptive approaches, I refer to the accounts in which the normative focus is on other moral claims for assigning responsibilities rather than vulnerability itself. From this line of argument, vulnerability itself is not a sufficient moral ground to point to responsibilities. If the “claim to protect vulnerable” exists, then it is on another “valid claim that some wrong should be avoided” such as basic needs or rights. Therefore, the notion of vulnerability helps us to consider how certain groups or individuals are more likely to be deprived of certain goods they have a claim for, and nothing more [22: 201, 35]. Nevertheless, the vulnerability approach and its dependency aspect also points to why there is a valid claim towards the assistance of a particular individual or group of people that will be discussed in the first part.

4 Brock [6] contends that the interests at stake for the populations in developing countries are significant enough to warrant limitations on the right to exit, but she simply assumes this line of thought without much theorizing on the right to exit and what it entails. Regarding the second point, she discusses and lists certain conditions for restrictive measures to be permissible. These conditions are that the coercive developing state should be legitimate, respecting human rights, showing good-faith efforts to sustain its vital institutions, and already investing in health worker training and institutional-development while still suffering from the mass exodus of skilled workers.

5 Labelling certain groups as unable to protect their interests (the conventional approach in research ethics) carry the risk of stereotyping, stigmatizing or unwarranted paternalism [28: 6]. However, in the context of protecting public health, I emphasize the need to have a non-stigmatized conceptualization of dependency relationship.

6 Depending on one’s sociocultural norms, expectations might be higher on adult children to care for their elder parents. Therefore, a communitarian argument might be another alternative to account for why adult children have demanding responsibilities towards their elder parents.

7 This is also where a global justice approach to issue might suggest that the developmental measures as such should be partly compensated by the developed countries who actively recruit health workers from the developing countries or on the basis of a more comprehensive and cosmopolitan responsibility model.

8 Brock uses the term”responsible, but poor” to describe the responsible governments of resource-poor developing countries [6: 60].

9 I only focus on care workers such as physicians, nurses and dentists, and exclude technicians and researchers for the sake of the debate.

10 By continuous care, I mean both (i) patients not being left without care for certain periods of time, and (ii) health workers not being regularly replaced by other health workers.

11 As mentioned before, ‘vulnerability’ is not treated as a moral trump card in this article to directly justify restrictions.

12 A non-restrictive alternative to coercive measures is a compulsory service contract that conditions the provision of state-funded tertiary education on an acceptance of a two-year compulsory service. Brock argues that the reasons why skilled workers themselves have responsibilities to assist should make us consider the compulsory service contracts a fair arrangement [6]. I agree that it is reasonable to ask a would-be medical student to temporarily relinquish her right to exit in exchange for the state-funded training she aspires to access. The vulnerability approach also brings about another line of defense for compulsory service contracts to be considered fair and reasonable. However, the terms, or the content, of the compulsory service programs, and how it treats the exchanged goods still require an additional reflection [9, 42].
